# Integrating socio-economic support into drug-resistant TB care to optimise treatment outcomes

**DOI:** 10.5588/ijtldopen.24.0534

**Published:** 2025-01-01

**Authors:** J. Limo, C. Pahe, I. Kathure, L. Ndungu, A. Mahihu, C. Mwashumbe, E. Mueni, H. Momanyi

**Affiliations:** ^1^ Population Services Kenya, Nairobi, Kenya;; ^2^ National Tuberculosis Programme, Nairobi, Kenya;; ^3^ Mombasa County Government, Mombasa, Kenya;; ^4^ Nairobi County Government, Nairobi, Kenya.

**Keywords:** adherence, mental health, person-centred care

## Abstract

**BACKGROUND:**

Drug-resistant TB (DR-TB) remains a public health concern in Kenya, with an estimated 2,500 individuals acquiring DR-TB annually. Despite significant progress in DR-TB management, the treatment success rate (TSR) in 2021 stood at 77%, falling short of the 85% target. This low TSR occurs amidst a complex range of treatment challenges, including psychosocial factors. The aim of this study was to assess the impact of integrating psychosocial and economic empowerment interventions into standard DR-TB care.

**METHODS:**

A convergent mixed-method approach was employed, involving interviews with 217 participants, including persons with DR-TB and key stakeholders, using a structured questionnaire. The study was conducted in Kenya’s two highest DR-TB burden counties between October and November 2023.

**RESULTS:**

The study found that 55% of respondents experienced psychological depression during treatment, with financial constraints identified as the primary trigger (70.6%). Only 49% of persons with DR-TB joined psychosocial support groups, and of these, 90% demonstrated improved treatment adherence. Additionally, individuals with DR-TB who participated in income-generating activities had a treatment adherence rate of 95%, compared to 88% among those not engaged in such activities.

**CONCLUSION:**

Patient-centred approaches involving socio-economic support systems are crucial in addressing treatment adherence barriers, thereby leading to improved treatment outcomes.

## RÉSUMÉ

**CONTEXTE:**

La TB pharmacorésistante (DR-TB) demeure un enjeu majeur de santé publique au Kenya, où l'on estime que 2 500 individus contractent cette maladie chaque année. Bien que des avancées notables aient été réalisées dans la prise en charge de la DR-TB, le taux de succès du traitement (TSR) en 2021 n'a atteint que 77%, ce qui est en dessous de l'objectif fixé de 85%. Ce faible TSR s'explique par un ensemble complexe de défis liés au traitement, incluant des facteurs psychosociaux. L'objectif de cette recherche était d'évaluer l'effet de l'intégration d'interventions d'autonomisation psychosociale et économique dans les soins standards de la DR-TB.

**MÉTHODES:**

Une approche mixte convergente a été adoptée, impliquant des entretiens avec 217 participants, incluant des personnes atteintes de DR-TB ainsi que des acteurs clés, à l'aide d'un questionnaire structuré. La recherche a été réalisée dans les deux comtés du Kenya présentant la plus forte prévalence de DR-TB, entre octobre et novembre 2023.

**RÉSULTATS:**

L'analyse a montré que 55% des répondants ont éprouvé des symptômes de dépression psychologique durant leur traitement, les difficultés financières étant reconnues comme le principal facteur déclencheur (70,6%). Seuls 49% des individus atteints de DR-TB ont intégré des groupes de soutien psychosocial, et parmi ceux-ci, 90% ont affiché une meilleure conformité au traitement. De plus, les patients atteints de DR-TB engagés dans des activités génératrices de revenus ont présenté un taux d'adhésion au traitement de 95%, comparativement à 88% pour ceux qui n'étaient pas impliqués dans de telles initiatives.

**CONCLUSION:**

Les stratégies axées sur le patient, intégrant des dispositifs de soutien socio-économique, sont essentielles pour surmonter les barrières à l'adhésion au traitement, ce qui contribue à l'amélioration des résultats thérapeutiques.

Multidrug-resistant TB (MDR-TB) is a major challenge to ending TB by 2035. According to the 2019 WHO Tuberculosis Report, the DR-TB incidence was estimated to be 1.3% among new TB cases and 4.6% among previously treated individuals globally.^1^ However, the WHO Tuberculosis Report 2022 reported an estimated 450,000 incident cases of MDR/RR-TB in 2021, representing an increase of 3.1% from 437,000 in 2020. The main reason for this increase is an overall rise in TB incidence between 2020 and 2021, largely due to the impact of the COVID-19 pandemic on TB detection.^2^ Additionally, an estimated 191,000 deaths occurred due to MDR-TB or rifampicin-resistant TB (RR-TB) in 2021. The WHO End TB Strategy (2015–2025) indicates that 48% of people with TB face catastrophic costs.^3^

In Kenya, drug-resistant TB remains a public health concern, according to National TB program annual report 2021 it was estimated that 2,500 people acquiring DR-TB annually.^4^ The country has adopted the WHO injection-free DR-TB regimen and utilises three care delivery models: facility, community, and isolation, with most patients following the community model of care. The drug resistance survey (DRS) conducted in 2015–2016 showed a prevalence of MDR-TB of 0.7% among new cases and 2.1% among retreatment cases. All individuals with DR-TB who were initiated on treatment had a TSR of 77%, with a death rate of 13%. TB disproportionately affects those aged 25–44 years, the most economically productive age group.^5^

The Kenya Programmatic Management guidelines provide operational guidance on the management of DR-TB. These guidelines define the structure of centres managing DR-TB, guided through discussions by DR-TB clinical management teams. The DR-TB management team is required to discuss new DR-TB cases before treatment initiation and meet regularly to address challenges during treatment, such as side effects, pregnancy, poor adherence, psychosocial barriers, and other issues. The management committee conducts home visits before treatment initiation. All doses of second-line drugs are to be observed by healthcare workers (HCWs) and confirmed as taken. Persons with DR-TB are monitored closely for clinical and bacteriological progress, the emergence of adverse drug effects, and appropriate actions taken as needed.^6^

The use of digital technology, such as SMS or phone calls, medication monitors, and video-supported treatment as a follow-up support system, has also proven effective. Patient and family support offered by MDR-TB treatment facilities includes food, transport, non-TB drugs, and investigations. Social support is provided directly to patients to ensure that assistance reaches those who need it most.

Despite progress in managing drug-resistant TB in Kenya, the TSR remains low at 77%, against a target of 85%. The death rate is 13%, and the loss to follow-up rate stands at 5.4%.^5^ The low TSR is attributed to complex treatment challenges, including psychosocial issues posed by the disease and the long treatment journey. According to the first Kenya TB cost survey, 86.4% of DR-TB patients experienced catastrophic expenditure, which is three times more than that of drug-susceptible TB (DS-TB) patients at 26.1%. Furthermore, more men (40.5%) lost jobs compared to women (24.6%), with 57.4% of DR-TB patients reporting job loss compared to 34.2% of DS-TB patients.^7^ Other challenges noted include food insecurity and relationship breakdowns, which were more common among DR patients than DS patients. This highlights the need to adapt and evaluate innovative solutions to address the current challenges of the DR-TB response.

## METHODOLOGY

The study employed a cross-sectional design using a convergent mixed-method approach, combining qualitative and quantitative methodologies collected simultaneously. The study was conducted in the counties of Nairobi and Mombasa (October–November 2023) in DR-TB treatment centres, where care implementation and management were carried out in 71 centres in Nairobi and 40 in Mombasa. The study utilised both quantitative and qualitative data collection methods. Active patients with DR-TB who were still on treatment and those enrolled in psychosocial support groups and income-generating activity groups were interviewed, and economic evaluations were extracted from investment records.

A sample size of 174 was used, proportionately distributed across the two geographical locations. Nairobi had a sample size of 111, and Mombasa had 63, based on the TB burden. The sample was further distributed among DR-TB patients in psychosocial support groups (91 individuals), income-generating activity groups (35 individuals), and those not enrolled in any group (48 individuals). Sampling methods included probability, purposive, and proportionate sampling. Cochran's formula was used to calculate the sample size, assuming a confidence level of 95%, a margin of error of 5%, and a target proportion of 50%. Data were cleaned using STATA analytical software (Stata Corp, College Station, TX, USA), and Do-files and syntax were stored for reference. Analysis was conducted using STATA 16, with visualisations done in Power-BI (Microsoft, Seattle, WA, USA).

### Ethical approval

The research permit was sought from NACOSTI (National Commission for Science, Technology and Innovation) and approved by Amref Health Africa, Ethics and Scientific Review Committee, Nairobi, Kenya (Protocol Ref: No. AMREF-ESRC/P1538/2023) approved on 27 October 2023. Informed consent was obtained from all subjects involved in the study.

## RESULTS

### Demographics of the interviewed respondents

**Figure 1.**
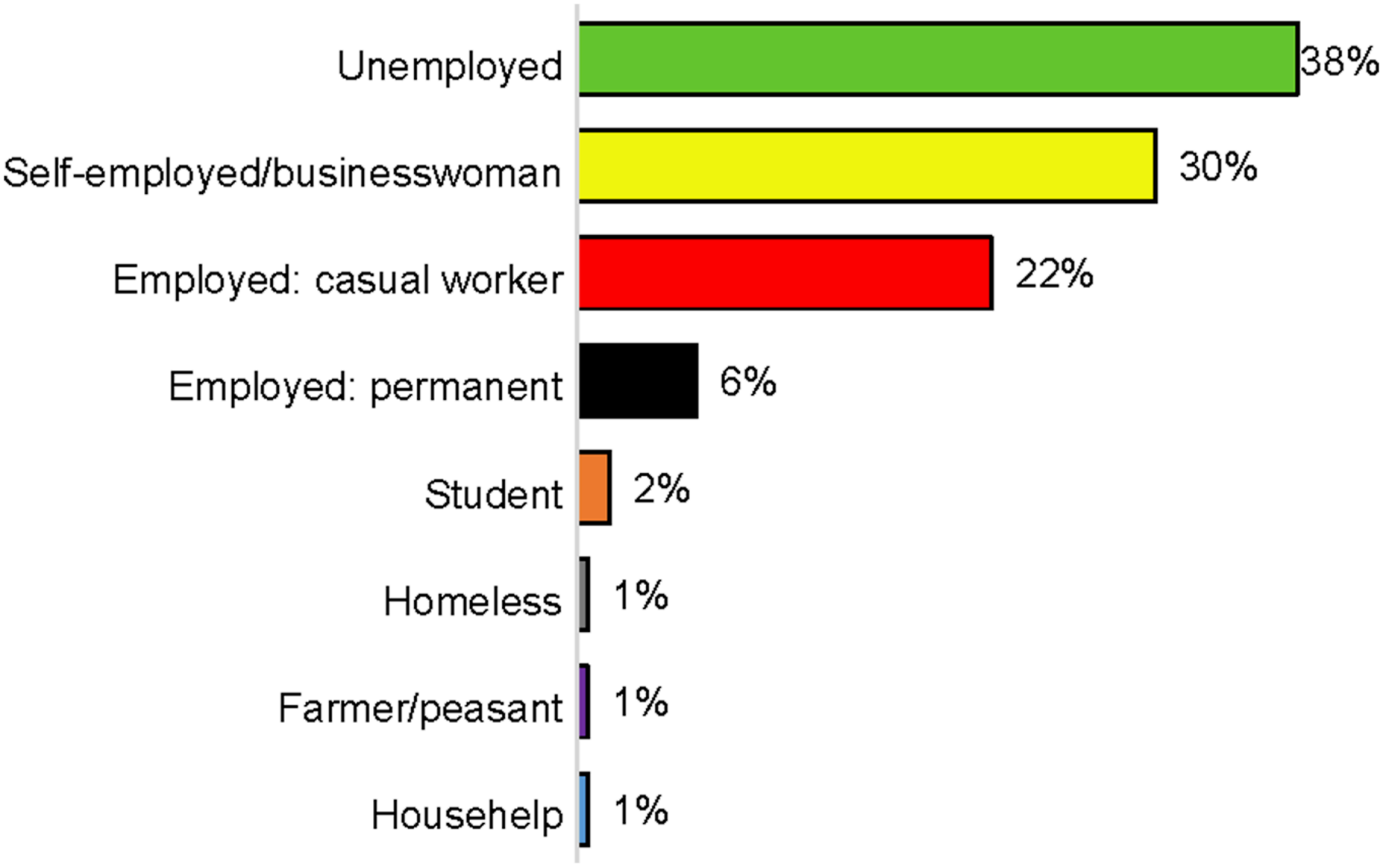
Patient occupation.

The response rate for the study was 94.8%, with 165 interviews achieved out of the target 174 respondents. Most participants (67%) were aged 25–44 years, while only 19% were aged above 50 years. The majority of respondents (45%) were married or living with their spouses, followed by 35% who were single. Most patients (38%) were unemployed at the time of the interview, with 30% self-employed. Only 6% had permanent employment at the time of the study. The details of patient occupations are shown in Figure 1. The majority of patients had no dependents at the time of the survey, with 36% and 38% having no minor or adult dependents, respectively. The average number of adult dependents was 1.7, while the average number of minor dependents was 1.2.

### Type of drug-resistant TB

**Figure 2.**
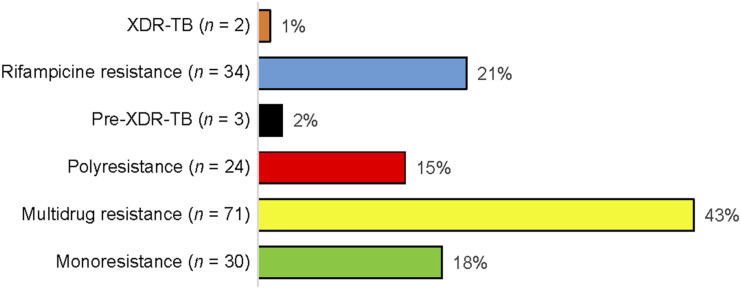
Type of drug-resistant TB. XDR-TB = extensively drug-resistant TB.

The majority of respondents (43%) were on MDR-TB treatment, followed by 21% with rifampicin resistance. Mono-resistance and poly-resistance were also prevalent at respectively 18% and 15%. Details of the types of DR-TB among respondents are shown in Figure 2. At the time of the interview, most patients (74%) were receiving facility-based care, a trend observed across counties, patient groups, and genders. Community-based care was utilised by 25%, while only two female patients from Nairobi were on the isolation model of care.

### Treatment adherence

Overall, 91% of respondents reported taking treatment without missing a dose. More females (94%) were adhering strictly compared to males (90%). Patients enrolled in income-generating activities (IGA) and psychosocial support showed better adherence than those not enrolled. Details of treatment adherence are shown in Table 1. Common challenges to TB medication adherence included peer pressure, pill burden, treatment costs, HIV-related stigma, alcohol use during treatment, lack of family support, and the perception of feeling better, which led some to discontinue treatment. Participants faced a significant pill burden, taking more than 10–12 tablets daily for 18 months. Male participants living alone highlighted nutritional challenges that impacted daily treatment adherence.

**Table 1.** Treatment adherence.

Taking treatment without missing a dose	Sex		Patient group			
	Female (*n* = 48)	Male (*n* 117)	Income-generating activity (*n* = 41)	Not enrolled (*n* = 43)	Psychosocial support (*n* = 81)	Total (*n* = 165)
No (*n* = 15), %	6	10	5	12	10	9
Yes (*n* = 150), %	94	90	95	88	90	91

### Treatment completion

By the time of the interview, 67% of respondents had completed their treatment successfully. Treatment completion was higher among patients from Nairobi and those receiving psychosocial support. Patients not enrolled in IGA or psychosocial support had the lowest treatment completion rate at 56%. Details of patient involvement in socioeconomic activities and treatment completion are shown in Table 2. Overall, 88% reported experiencing side effects during treatment, with skin burns and weakness being the most common. Side effects were reported more frequently by patients from Mombasa, females, and those engaged in socio-economic activities. Patients in psychosocial support programmes reported fewer side effects compared to others.

**Table 2.** Treatment completion.

	Sex		Patient group			
Completed treatment	Female (*n* = 48)	Male (*n* 117)	Income-generating activity (*n* = 41)	Not enrolled (*n* = 43)	Psychosocial support (*n* = 81)	Total (*n* = 165)
No (*n* = 54), %	35	32	32	44	27	33
Yes (*n* = 111), %	65	68	68	56	73	67

### Social economic support groups

Most respondents (88%) were enrolled in psychosocial support groups, with 59% participating in IGA, while some were enrolled in both. More females (69%) were enrolled in social support groups than males (54%). Interviews indicated that participation in psychosocial groups provided emotional and practical benefits throughout the treatment journey. While positive impacts were noted across both Nairobi and Mombasa, variations existed in the frequency of meetings and oversight. The common benefits included financial aid, emotional support, and a collective commitment to growth and well-being during DR-TB treatment.

### Income-generating activities

Overall, 31% of those enrolled in social support groups had IGA at the time of the study. Half of the respondents from Mombasa were enrolled in an IGA, compared with only a quarter in Nairobi. There were slightly more males (33%) engaged in IGA than females (27%).

### Experiencing stress/depression

Overall, 55% of respondents reported experiencing stress or depression at the time of the study. Patients particularly affected by higher levels of stress and depression were from Mombasa, especially males and those involved in IGA.

### Causes/triggers of stress/depression

Financial constraints were reported as the most significant contributor to stress and depression among respondents, with 70.6% identifying it as a trigger. Lack of employment was the second most frequently cited factor, affecting 42.5% of respondents. Other contributors included food insecurity (27.5%), the burden of frequent medication intake (26.1%), childcare responsibilities (19.0%), and sleep disturbances (13.7%). Several actions were taken by respondents when experiencing stress or depression, including seeking psychological counselling, oversleeping, using drugs and other substances, seeking support from friends, and consuming alcohol.

### Patient satisfaction

Overall, most respondents were satisfied with their treatment experience, scoring an average above 4 points on a five-point scale for clinical review meetings, adherence counselling, home visits by adherence counsellors, and financial incentives received during treatment. However, satisfaction regarding peer income-generating groups and peer group support was lower, scoring 3.1 and 3.8, respectively. Details on patient satisfaction are shown in Table 3.

**Table 3.** Treatment process satisfaction rating.

Satisfaction statements	Average satisfaction score	Average rating %	Satisfied score %	Unsatisfied score %
Peer support group	3.8	77	13	24
Adherence counsellors	4.3	87	22	7
Adherence counsellor home visits	4.3	86	19	9
Income-generating groups	3.1	61	5	38
Clinical review meetings	4.5	91	23	4
Financial incentive support you receive during treatment	4.2	84	18	19

### Patients' recommendations for improvement

Slightly above 50% of DR-TB patients agreed that treatment was challenging and called for additional measures to ease their difficulties. Given that treatment made them weak and unable to do heavy work, they recommended monetary support during treatment to cushion economic challenges. While IGA were considered helpful, they faced challenges, particularly related to mismanagement. Patients suggested that IGA leaders should be independent and not part of the patient group to minimise mistrust and resource mismanagement.

## DISCUSSION

The socio-economic burden brought about by DR-TB service delivery and management highlights the need for an adequate assessment to better utilise innovative approaches in improving DR-TB management. This study assessed several innovative approaches, including engaging counsellors as ‘treatment buddies’ to support treatment adherence, establishing psychosocial support groups for people with DR-TB, and supporting persons with DR-TB to participate in IGA to facilitate financial recovery and improve adherence. A study in Uganda found that improving treatment outcomes for persons with DR-TB requires addressing socio-economic determinants alongside general programmatic improvements. Institutionalisation of treatment enablers, such as food provision, transport support, and micro-financing mechanisms, is particularly important in poverty-stricken areas and should be a priority for health authorities.^8^

### Patients' experience on the DR-TB treatment journey

In this study, most respondents, both patients and caregivers, demonstrated good awareness and understanding of TB and drug-resistant TB. They associated traditional 6-month treatment with normal TB, while recognising that DR-TB required 12–18 months of treatment. They also demonstrated a good understanding of clinical symptoms, such as sudden weight loss, persistent coughing, night sweats, and breathing difficulties. These findings are consistent with a study in China, which found that 92% of patients understood pulmonary TB transmission.^9^ In this study, the facility-based model of care was the most utilised (74%), followed by community-based care (25%) and isolation (1%), with similar patterns across different regions, patient groups, and genders.

The majority (91%) of respondents reported taking treatment without missing a dose, with 94% noting improvement in their condition. However, their reported levels of improvement varied: 73% reported great improvement, 21% reported some improvement, 2% reported no improvement, and 4% were undecided. These results contrast with a study in China, which reported that only 66.4% of pulmonary TB patients were adherent.^9^

Respondents also reported facing several challenges during their DR-TB treatment journey. Of those interviewed, 88% experienced side effects, with the majority reporting skin burns and general weakness. They also faced a significant pill burden, taking 10–12 tablets daily for 18 months. Male participants living alone highlighted nutritional challenges, which negatively impacted their daily lives. High treatment demands, such as daily facility visits or home visits from healthcare providers, led to economic challenges and job loss for some patients. Other common challenges to TB medication adherence included peer pressure, treatment costs, HIV-related stigma, alcohol use during treatment, lack of family support, and the perception of feeling better, leading to treatment discontinuation, particularly among women.

### Impact of socio-economic activities

***Psychosocial support activities:*** The majority (55%) of respondents reported experiencing psychological stress or depression during treatment, with financial constraints (70.6%), including lack of a job (42.5%), as the major triggers. Frequent medication intake was also cited as a trigger for stress or depression (26.1%). Other factors, such as exhibiting TB symptoms and stigma from neighbours and community members, also contributed to stress or depression (6.5%). Respondents varied in their responses to psychological challenges: 24.3% sought psychological counselling, 10% used drugs, 8.6% took alcohol, and 0.7% admitted to self-harm. This rate of depression is higher than the findings of a study in India, which reported a 16.2% prevalence of depression among MDR-TB patients.^10^

Engagement of adherence counsellors was considered essential by both patients and opinion leaders as a psychosocial support strategy. Counsellors acted as ‘treatment buddies’, supporting DR-TB patients in adhering to treatment by conducting baseline mental health and risk assessments and repeating these assessments every 3 months using Patient Health Questionnaire-9 (PHQ-9) and other risk assessment tools: 83% of the persons with DR-TB were visited by adherence counsellors at their homes at least once a week (41%), monthly (28%), twice a month (13%), and once in 6 months (18%). Home visits were deemed vital for assessing patients' conditions, providing outreach services, conducting contact screening, and facilitating communication with family members; however, adherence counsellors faced challenges in the home visit activities, including patients unwilling to receive assistance, insecurity due to patients residing in dangerous environments like slums with gang violence, street families, and drug-related users.

This aligns with another study on Outcome Optimization for Patients with Drug-Resistant TB, which highlighted the fact that improving DR-TB outcomes may need a comprehensive plan that includes psychological assistance. Frequent patient contact and better nutrition promote greater treatment adherence, and home visits and help from psychologists may further assist persons with DR-TB and, ultimately, result in their cure. In that study, 88 of the 100 persons with DR-TB who received the entire care package had successful outcomes, a considerable improvement over the previous cohort; 4% of the attempts failed, while 7% of persons with DR-TB passed away during therapy. Significantly, just one patient was transported out, and there were no incidents of loss to follow-up.^11^

Adoption of peer-peer psychosocial support groups for patients to enhance treatment adherence and the provision of patient support are key to better treatment outcomes. A study by Rama et al. found that the TSR was significantly higher in the supported group (65% vs 46.0%). This is in line with this study's findings, where 73% of patients who participated in support groups had better treatment outcomes: 58% of the respondents reported being in at least one social support group, and 2% were in more than two groups; however, 40% reported that they were in none of the groups. A study conducted in South India on the challenges faced by DR-TB patients and their caregivers found that both patients and caregivers were concerned about the absence of supportive care from family members, as well as a lack of empathy and intangible social support.^12^

In a similar study, the involvement of caregivers was highlighted as a crucial support strategy. Caregivers played an essential role in supporting patients with DR-TB by encouraging adherence to medication, providing food, accompanying them to health facilities, and offering emotional support during challenging times throughout treatment. These strategies were effective largely due to the strong emotional connection between patients and caregivers, especially since many patients with DR-TB experienced neglect or stigma from family members.

***Income-generating activities****:* According to a study conducted in Uganda, initiatives targeting poverty and other social determinants of health have the potential to accelerate progress towards End TB targets by protecting against TB-associated catastrophic costs and reducing TB incidence and mortality over the long term. In this study, a majority of persons with DR-TB (38%) were unemployed, while 30% were self-employed, 22% were in casual employment, and 6% were in stable employment. Only 31% were engaged in IGA, 60% were not in any, 8% did not know about them, and 1% did not respond.^8^ Participants involved in IGA pursued a variety of objectives, all of which aligned with the overarching aim of balancing economic empowerment with the effective management of DR-TB treatment. The active DR-TB patients involved in IGA showed a treatment adherence rate of 95%, compared to 88% among those not engaged. Similar findings were reported by Wen et al. who noted an encouraging improvement in the TSR when material support was integrated into social support packages.^13^

Despite their positive impact, those in IGA faced several challenges, including financial mismanagement of the seed grants awarded to some groups. Successful income-generating groups benefited from business skills training and financial management training, which were suggested to reduce financial mismanagement. The business activities encouraged persons with DR-TB to work together towards a common livelihood goal. Caregivers noted that their patients were socially and emotionally encouraged to participate in these activities.

### Study limitations

One notable limitation of this study is that the data collection focused exclusively on the urban population. Therefore, the findings and conclusions drawn from this research may not be entirely applicable or generalisable to rural populations. Rural areas often have distinct socio-economic, cultural, and infrastructural differences, which could influence health behaviours and outcomes differently compared to urban settings. Consequently, the study's conclusions should be interpreted within the context of urban environments, and caution should be exercised when attempting to extrapolate these results to rural populations without further specific research in those areas. Future studies encompassing both urban and rural populations would provide a more comprehensive understanding of the topic under investigation.

## CONCLUSIONS

The study reveals that, although patients and caregivers demonstrate strong awareness of DR-TB, the treatment journey is fraught with psychological, economic, and social challenges. Effective TB treatment requires a holistic, patient-centred approach that integrates adherence to medication with psychosocial and socio-economic support systems. These elements play a key role in addressing barriers to treatment adherence and increasing the survival rate among persons with DR-TB, ultimately leading to better treatment outcomes. Addressing these socio-economic determinants must be paired with improvements in the general programmatic performance of DR-TB treatment.
